# Fracture of Jammed Colloidal Suspensions

**DOI:** 10.1038/srep14175

**Published:** 2015-09-16

**Authors:** M.I. Smith

**Affiliations:** 1 School of Physics, University of Nottingham, University Park, Nottingham, UK

## Abstract

Concentrated colloidal suspensions display dramatic rises in viscosity, leading to jamming and granulation, with increasing shear rate. It has been proposed that these effects result from inter particle friction, as lubrication forces are overcome. This suggests the jamming of concentrated colloidal suspensions should exhibit some shared phenomenology with macroscopic granular systems where friction leads to two different types of jammed state. Here we show that transient rheological measurements can be used to probe the processes of granulation in concentrated colloidal suspensions. Our results support the idea that frictional contacts are created between jammed particles. The jamming behaviour displays two qualitatively different regimes separated by a critical strain rate with qualitatively different types of fracture/break up behaviour. In the lower strain rate regime, it is found that vibrations can be used to control jamming and granulation, resulting in a flowable fluid.

Particulate suspensions at high volume fractions are present in a range of industrial pastes and fluids^1^. Understanding and controlling jamming in particle fluids is therefore important in the design of pumping and dispensing processes. Whilst granular fluids have interactions dominated by frictional contacts, colloids are hydrodynamically lubricated by films of solvent. However, in concentrated colloidal suspensions, above some critical applied stress, it has been observed that there is a discontinuous jump in the shear viscosity^2^. Recent studies have linked this discontinuity in viscosity to the stress required to overcome lubrication films, resulting in frictional contacts between particles^2^,^3^,^4^. This implies an important conceptual connection with granular materials.

Coining the term *fragile matter*, Cates *et al*^5^ considered an idealised material which contained force chains – a network of touching particles which bear a disproportionate fraction of the stress applied to the sample. One would expect such materials to be elastic in the same direction as the load which formed the force chain network. However, the network would change irreversibly when subjected to small forces in other directions. Since the orientation of force chains may be anisotropic, the three dimensional nature of a jammed sample becomes significant^6^.

Whilst previous studies have considered the transition from a liquid to jammed granules^7^,^8^, here we focus on the jammed state itself. We show that concentrated colloids display two different types of jamming, with changes in fracture mode. We also find that one of these types of jamming can be suppressed with small amplitude vibrations.

## Basic phenomena

Our experiments use a shear rheometer with parallel plates, (D = 6 mm, initial gap = 2 mm). Between the plates is sandwiched a high volume fraction (φ ~ 0.62–3) colloidal fluid of ~2 μm diameter particles (PMMA-PHSA) in Octadecene (see supplementary information). The top plate of our shear rheometer, is moved upwards at a fixed velocity, like an extensional rheometer, although at smaller velocities (V ~ 0.001–5 mms^−1^). The use of a shear rheometer to perform extensional measurements enabled us to measure the resistive forces as the plates are separated using the instrument’s normal force sensor. Each experiment was also filmed with a camera (Point Grey Flea 3, resolution 496 × 984, 0.1–50 fps).

**Figure 1:**
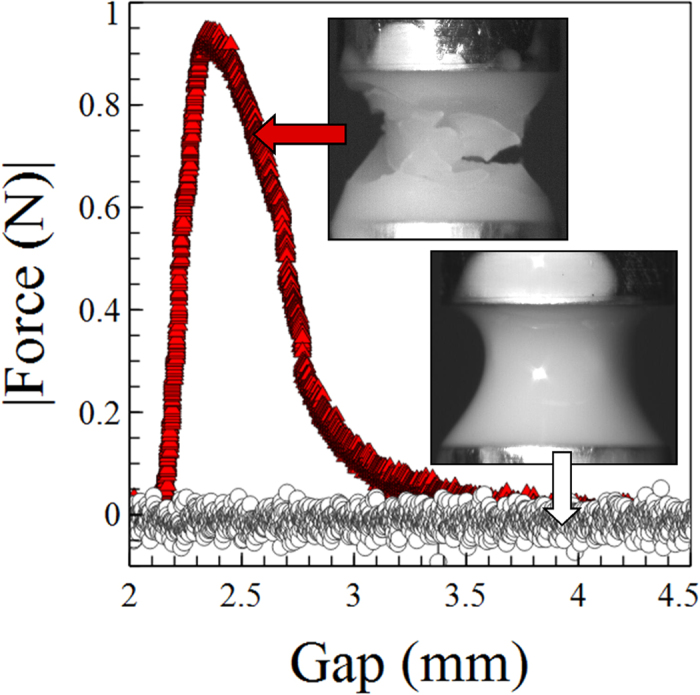
Example force curves for a PMMA-PHSA suspension of ~2 μm particles (φ ~ 0.631). The upper plate is moved upwards at v = 0.03 mms^−1^. Experiments were performed with (O) and without (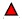) vibration of the bottom plate (f = 300 Hz, Acc. = 30 ms^−2^). Insets show images of the vibrated (bottom) and unvibrated (top) samples.

When the upper plate is moved slowly we observe that the suspension flows smoothly as a liquid. Whilst the fluid remains pinned at the end plates the central region, thins, necks and undergoes a liquid type break-up behaviour. However, above a critical velocity the sample jams and fractures, resulting in the formation of granules^8^. Figure 1 shows a typical force curve measured during an experiment (φ ~ 0.631, v = 0.03 mms^−1^). As the plates move apart there is an initial movement ~0.1 mm before any force is measurable. Following this the force rises fairly linearly with plate separation to a maximum. The fluid surface changes from glossy to matt quite suddenly just prior to the maximum in each force curve, although *after* the strong rise in force has been detected. The change of the liquid texture from glossy to matt is often associated with a dilatant, system spanning network of force chains^7^. However, clearly significant resistance to flow occurs prior to this indicating significant shear thickening. Closely following this change in the appearance of the surface, fractures propagate. New fractures continue to appear as the now jammed granule breaks apart, decreasing the measured force. The granules then remain jammed indefinitely (see supplementary information).

**Figure 2:**
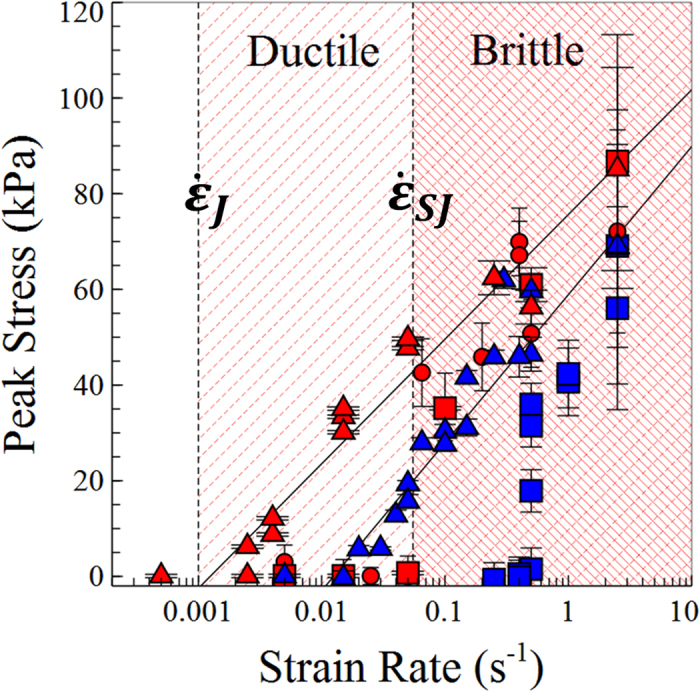
The effect of vibrations on jamming. The peak force is plotted for two different volume fractions ϕ ~ 0.631 (red), ϕ ~ 0.625 (blue). At low strain rates, without vibrations (Δ) samples flow, corresponding to a negligible force. Above some critical strain rate (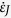) the samples jam and fracture. In samples where the bottom plate is vibrated (○ = 300 Hz, □ = 100 Hz) the samples are observed to flow like a liquid at strain rates in the range (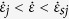 ). However, at strain rates above 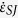 these samples jam. The peak force measured above 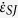 quickly approaches the same value as the unvibrated samples.

Force curve measurements were performed on two different volume fraction samples (φ ~ 0.631 and 0.625) at different plate velocities (v ~ 0.001–5 mms^−1^). Extracting the peak force (F_max_) from these curves, we converted our measurements to approximate stresses (σ_max_ ~ 4F_max_/πD^2^) and strain rates (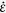 ~ v/l_0_), where D is the plate diameter and l_0_ the initial separation between the plates. Figure 2 shows how the peak stress varies with the applied strain rate. At low strain rates, in samples that display liquid-like flow behaviour, the force is too small to be measured. At a critical strain rate 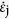 we observe the onset of jamming. As the strain rate is increased above 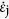 the measured peak in the force rises. However, it should be noted that the stress dependence on the strain rate is surprisingly weak (N.B. the stress axis is a linear not log scale).

Hartley *et al.*^9^ showed that in a 2D system of frictional discs the stress upon shearing, varied logarithmically with shear rate. This increase in stress was caused by the irreversible rearrangements of the force chain network. In Fig. 2a similar strain-rate dependence is observed, with the maximum value of stress of the jammed state, fitted by the form σ ~ A log (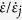) (see Fig. 2). We also note that the stress scale far exceeds what might be expected for lubricated contacts (The shear rheology for a similar system of slightly smaller particles at very high volume fractions was measured in^8^ resulting in stresses several orders of magnitude lower). This suggests an additional contribution to the viscosity not present at lower stresses. The indefinite stability of the granule after the measurements, also suggests that these contacts cannot be purely transitory, maintained by lubrication forces, since this requires relative motion of the particles^4^. Rather the particles must maintain static contacts^10^. Finally, the fact that the jammed state (at which all flow towards the end plates is visually observed to cease) occurs near to the maximum of the force curves raises a question about the relationship to prior stages of the flow. The smooth increase in force implies some continuity in mechanism between the initial resistance to flow and the peak stress of the jammed state. These combined observations suggest both discontinuous shear thickening and jamming of “hard sphere” colloids can be understood to depend on frictional contacts between particles.

From Fig. 2 lower bound of the maximum jammed stress σ_max_ is ~88 kPa. This is comparable to the maximum capillary stress for our particles ~5.3γ/a = 172 kPa^11^ where a is the radius of the particles. Recently, the capillary stress scale for a particle fluid during droplet pinch off was calculated to be ~αγ/a4√3 (~4.6 kPa for our system)^12^. This latter case, in which the flow is not forced by movement of the rheometer plates, provides a sensible lower bound for the jamming stress if capillary forces are important. The lowest measured stresses in Fig. 2, for which jamming occurs, are comparable to this figure. Since jamming appears to occur within this range, this suggests capillary forces between particles at the liquid-air interface set the stress scale, in agreement with the commonly held model for colloidal granules^7^,^10^. In the jammed network of a quasi-stable granule this confining stress must be balanced by internal forces within the granule, namely contacts between particles.

Given the value of the stresses measured, one might question whether the stabilising layer of grafted polymer chains on the colloidal particles remains intact. Following a number of experiments at high strain rates, we took small quantities of the jammed granule and diluted in solvent to low volume fractions. The samples were agitated purely by turning the vial gently end over end. The colloid dispersed easily. Observation of the solution under a microscope showed that the colloids were not stuck together. This suggests that no permanent damage is being done to the steric layers on the colloids by the experiment, and consequently observed phenomena do not arise from adhesion of degraded particles.

## The stability of jammed states

Once created, colloidal granules exhibit a marked bi-stability^7^. Apparently stable static granules will return to a flowable state with a gentle poke from a spatula. However, we observed that *during* a stretching experiment, if the surface of a sample was perturbed with a spatula then the sample failed to jam. Even, if the surface was allowed to begin to turn matt and fracture a gentle poke of the air-liquid interface in one position was sufficient for the entire sample to revert back to a liquid. This also apparently “reset” the timescales, since following this perturbation the sample would remain flowable for a period before the measured force again began to rise. However, we noticed that as the strain rate was increased the sample apparently became more resistant to this relaxation process.

To investigate this behaviour in a more refined way we designed a custom bottom plate for our rheometer which could be vibrated vertically. Small sinusoidal vibrations (freq 100 or 300 Hz, acceleration in both cases ~30 ms^−2^, amplitude ~80 or 8 μm) were applied continuously whilst moving the upper plate. Figure 1 shows a force curve (○) and image for a sample of identical composition (φ ~ 0.631, v = 0.03 mms^−1^) to that discussed earlier, where the bottom plate was vibrated. Despite moving the upper plate at a strain rate above the critical value required to produce jamming (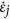) we observe liquid-like flow. Supplementary video 1 shows two samples under the same conditions, with (right) and without (left) vibrations. The vibrations act to suppress jamming.

Figure 2 quantifies this modification of the jamming transition. Plotting the peak stress of the measured force curves, it is clear that by applying a vibration to the bottom plate, jamming is suppressed even at strain rates an order of magnitude above 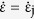. However, as the upper plate velocity is increased still further above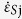 a higher strain rate threshold (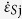), we again observe jamming and granulation. Intriguingly, however the peak forces measured during this higher strain rate region quickly approach that of the un-vibrated sample. It is not therefore simply that the vibrations are gradually becoming less effective, but symptomatic of a crossover in behaviour. To assess how generic this feature is we mapped the transition in detail at two different frequencies and constant acceleration (100 Hz □ & 300 Hz O, acc ~ 30 ms^−2^). We also performed isolated tests at different, frequencies, amplitudes and accelerations at strain rates above and below 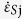. At frequencies above 500 Hz (acc ~ 30 ms^−2^) we saw a shift of this transition to lower plate velocities. However, we observed that the lower part of the sample relaxed like a liquid, whilst the upper part became lumpy and thickened, indicating that the vibrations do not penetrate to the upper part of the fluid. The failure of the vibrations to penetrate through the entire fluid column effectively limited the range of vibration conditions which could be meaningfully studied. We did however observe similar relaxation behaviour (with the same critical strain rate 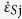) when the amplitude was varied at a fixed frequency of 100 Hz. However, as the amplitude (and hence acceleration) was reduced we again encountered issues to do with penetration of the vibrations through the fluid column. From the limited range of feasible frequencies, amplitudes and accelerations studied, it appears that provided the vibrations are sufficient to propagate through the column of fluid there are no changes in the position of the transition 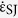. We also found that using roughened plates, jamming could be suppressed by torsionally oscillating the rheometer plate during extension measurements. Together with our earlier observation that a spatula can be used to suppress jamming, this suggests the details of the perturbation are relatively unimportant.

We also repeated our experiments at a lower volume fraction (φ ~ 0.625). This shifted the flow and jamming behaviour of both vibrated and unvibrated samples to higher strain rates. However, 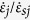 the approximate ratio of 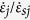 (the ratio of the critical strain rates required for jamming in the unvibrated and vibrated samples) remained constant at ~0.02. It is also notable that if we plot σ(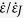) for both volume fractions we observe a collapse of all the data onto two curves: those with and without vibrations (see Supplementary Fig. 1). 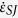 occurs at approximately the same stress in both volume fractions studied. It would be interesting, as well as useful for design of industrial processes, to know if this stress scale applies to a wider range of volume fractions. Unfortunately, the acceleration of our instrument (50 mms^−2^) prevents us from studying lower volume fraction samples.

**Figure 3:**
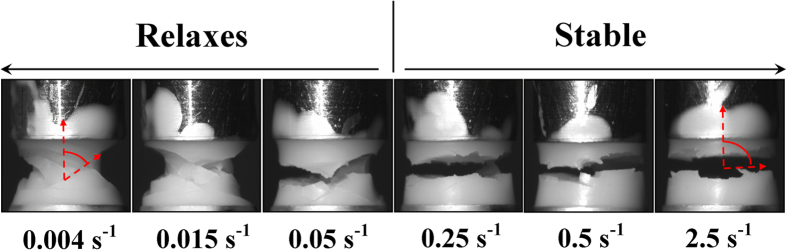
Images of the fracture of ϕ ~ 0.631 PMMA-PHSA colloidal suspension at the strain rates indicated without vibrations. The fracture planes (examples of which are highlighted in red) rotate from ~40–60° to ~90° at the same strain rate at which vibrated samples no longer relax.

To understand the significance of this sensitivity to vibration we performed further analysis on our *unvibrated* samples, looking for changes in behaviour located at the strain-rate 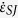. Figure 3 shows unvibrated samples just after fracture has occurred. Samples with strain rates 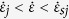 show fracture planes oriented at angles ~60–40° to the vertical which is probably related to the direction of maximum resolved shear stress (~45° for amorphous samples^13^). However for 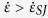, the same critical strain-rate (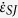) at which vibrations no longer unjam samples, the fracture surfaces become horizontal. This rotation of the fracture plane with increasing strain rate has also been observed in bulk metallic glasses^14^ and transient polymer networks^15^. Our samples are at volume fractions above the glass transition, and the jammed state could be thought of as a transient network. We would therefore expect to see some comparable behaviour.

Theoretical work by Furukawa *et al.*^14^ studied the response of glassy materials to a suddenly imposed stress. Without making connections to a microscopic picture, they examined the spatio-temporal evolution of density or composition fluctuations and the resultant breakup of the system. The growth of volume fraction fluctuations preceding the jamming transition has been demonstrated in different geometries for concentrated suspensions of non-Brownian colloids^16^,^17^,^18^,^19^. Concentration coupling, in which small volume fraction fluctuations lead to local shear gradients which in turn lead to larger variations in volume fraction has also been observed^20^ suggesting Furukawa’s work should be applicable to our experiments. Furukawa *et al.* predict three break up regimes at different strain rates. Low strain rates undergo “liquid type” break up which involves necking of the flow. Intermediate strain rates are predicted to undergo “viscoelastic type” break up, resulting in fracture along a 45° angle to the axis of elongation. Finally, at high strain rates “solid-type” break up is expected, which results in fractures at 90° to the same axis. This agrees with our results in Fig. 3. Whilst the model does not connect explicitly to the microscopic dynamics, in addition to the liquid flow regime at low strain rates, it suggests two types of qualitatively different jammed regimes with different instability/fracture mechanisms. Firstly, a viscoelastic instability which arises from the volume fraction dependence of the structural relaxation time, a purely dynamical phenomenon. Secondly, a solid-type instability connected to the volume fraction dependence of the elastic modulus, which is of static or geometric origin^14^.

**Figure 4:**
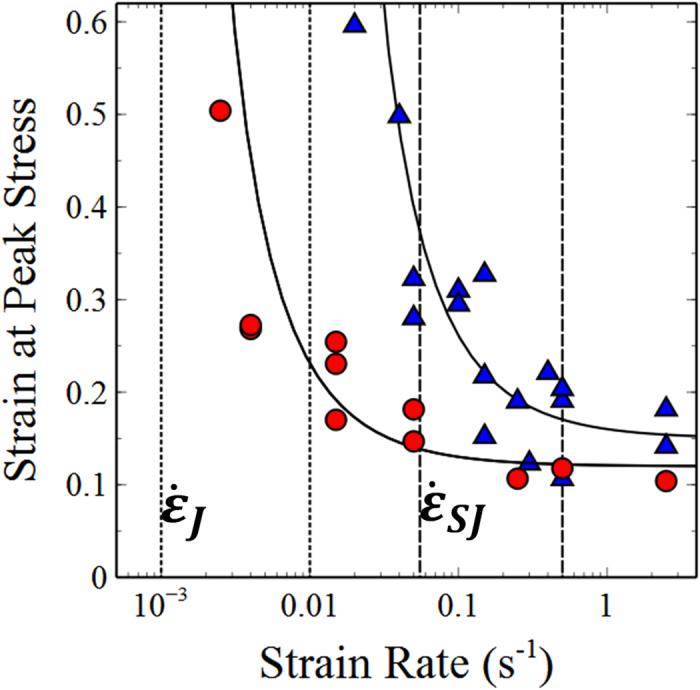
Strain at the peak stress for different strain rates, ϕ = (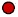) 0.631 and (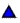) 0.625. The data is fitted to the equation 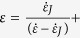 *ε*_elastic_ where 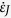 is determined from Fig. 2. The dotted lines indicate 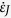 and 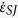 for both volume fractions.

Figure 4 shows how the strain measured at fracture varies with strain-rate. For both volume fractions studied, the sets of data exhibit a strong divergence at the strain-rate 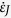 (small dashed lines) at which the samples first jam and fracture. More interestingly we observe that the strain (at which fracture occurs) appears to saturate at a finite value. The curves approximately level off at a strain-rate of 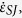 (large dashed lines).

The differences in fracture plane suggest an explanation for the observed behaviour. At high strain-rates samples undergo a brittle or purely elastic mode of fracture whilst at lower strain rates the samples undergo a ductile or viscoelastic mode of fracture. The strain at fracture (ε_Fracture_) should therefore be divisible into a plastic (ε_Plastic_) and an elastic (ε_Elastic_) contribution. In simple terms this plastic strain arises from a competition between structure formation given by the excess strain rate (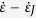) which causes shear thickening, jamming etc and the maximum rate for structural relaxation 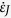, by which the sample reorganises in response to internal stresses^16^. The elastic contribution should however be rate independent and therefore dominates as the rate increases due to the lack of time for structural relaxation processes to take place. Summing these contributions the simplest form for the strain at fracture should be given by:


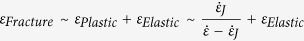


Figure 4 shows fits of this equation with one adjustable parameter *ε*_*Elastic*_ which has values of ~0.15 and 0.13 (±0.01) for volume fractions of 0.631 and 0.625. It is clear that the plastic contribution becomes small at strain rates above 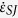 (long dashed lines Fig. 4) confirming the existence of a crossover in break up behaviour.

These observations and experiments lead to a key result in this study. That is, that there exist two qualitatively different jammed states for a colloidal granule. Whilst the lower strain rate jamming is fragile to perturbations and subject to plastic rearrangements, the higher strain rate shares some characteristics more in common with a conventional elastic material. These states are qualitatively different.

The existence of two types of jammed state, at different stress scales, was demonstrated by Bi *et al.*^21^ who analysed the forces on a collection of 2D perspex discs. This enabled them to connect the macroscopic behaviour to the underlying force chain network. At a threshold stress it was observed that the network transitions from an unjammed state to a ‘fragile’ jammed state. This fragile jammed state, which requires frictional interactions, exhibits an anisotropic force chain network which is strong along the compression direction but weak in all other directions. At still higher stresses another transition occurred, to a ‘shear jammed’ state, characterised by an isotropic force chain network. Recent simulation work of shear thickening colloidal fluids also supports the idea that the isotropy of force chain networks should change with shear rate in non-Brownian colloids.^22^

In our own work, the proposed force chain microstructure would be expected to develop to resist the applied stresses. At low strain rates, there is a breaking of the granule’s macroscopic symmetry. This implies an anisotropic stress distribution which would lead to an anisotropic network of force chains. At higher strain rates there is macroscopic symmetry leading one to expect a qualitatively different network of force chains. Our two jammed states, consisting of frictional contacts and different force chain distributions appear to be similar to the results of Bi *et al.*

This conclusion is strengthened when one considers the effect of vibrations. Unjamming of jammed colloidal samples was observed during flows of colloid from a needle^23^. Vortices created at the outlet of a needle unjammed the sample by applying a force in a different direction to the load which created the initial jam. In the current study, when the bottom plate reverses direction load bearing particle contacts are pulled slightly apart without compression of force chains in an alternate direction to prevent subsequent relaxation. Even small movements of a single load bearing particle alter the wider network. In contrast if the deformations of the network are elastic (and isotropic) in nature each cycle of the bottom plate returns particles to the same network configuration. This may explain why the vibration behaviour changes at or near the elastic limit, since even minimal structural relaxations can influence the macroscopic stability of the particle network.

## Additional Information

**How to cite this article**: Smith, M.I. Fracture of Jammed Colloidal Suspensions. *Sci. Rep.* **5**, 14175; doi: 10.1038/srep14175 (2015).

## Supplementary Material

Supplementary Information

Supplementary Movie 1
