# Supplementary material for: Fracture of Jammed Colloidal Suspensions
Source: Sci Rep. 2015 Sep 16;5:14175. doi: 10.1038/srep14175 (PMC4570995; doi:10.1038/srep14175)
Supplement: Supplementary Information [file srep14175-s1.pdf]

Supplementary Information

Fracture of Jammed Colloidal Suspensions

M.I. Smith

School of Physics, University of Nottingham, Nottingham, UK

e-mail: mike.i.smith@nottingham.ac.uk

Particle Size and granule stability

Following fracture the samples in the current study were found to remain jammed indefinitely. Testing this with a simple experiment we found it was possible to produce a granule and then having waited 24 hours convert it back to a flowable state with a small prod of a spatula. This is notably different from work conducted in ref 10 where samples fractured and then slowly returned to a liquid state. Here similar experiments were performed at a wide range of volume fractions, the highest of which are comparable to the present study. However, the particles used were smaller (~ 1.2μm as opposed to nearly 1.85μm). Indeed in unpublished work we found that it was not possible to produce a granulated sample in this way, with particles of ~700nm diameter, presumably due to the larger contribution of thermal noise with decreasing particle size. This seems to indicate an increasing jamming and stability of colloidal granules with increasing particle size.

Supplementary figure 1

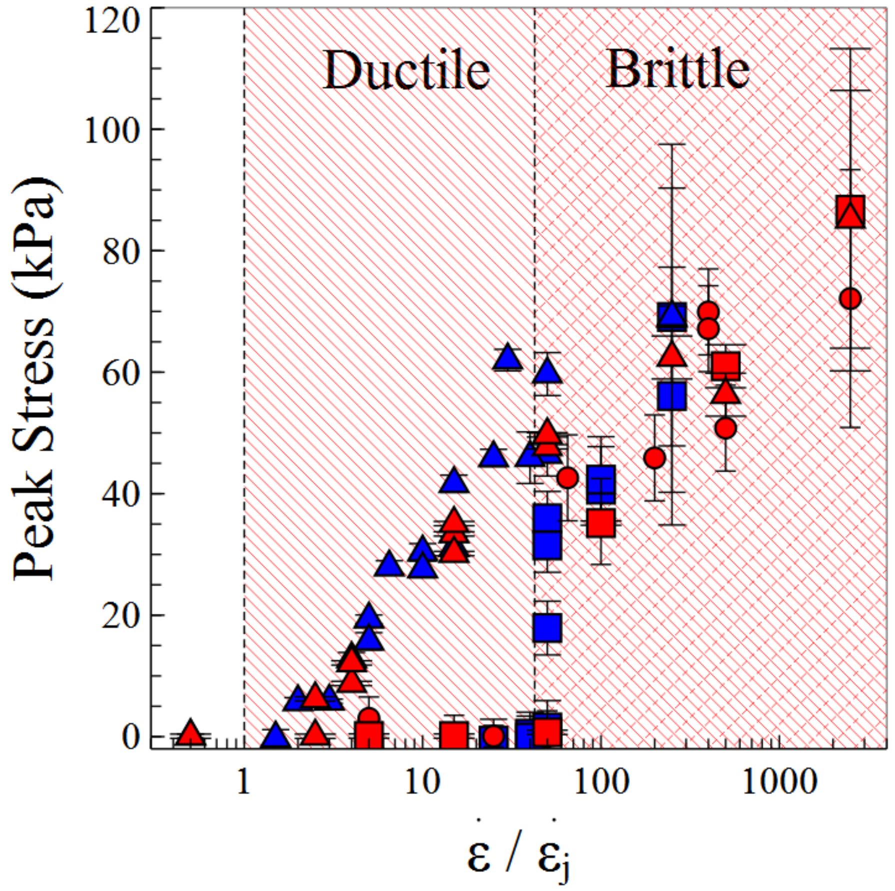

Supplementary figure 1: Scaling the data in figure 2 by the critical strain-rate required for jamming ($\dot{\varepsilon}_J$) results in a collapse of the data onto two curves: those with and those without vibrations.

Methods:

Sample preparation and Loading Protocols:

Experiments were performed with poly(methyl-methacrylate) spheres (diameter ~ 1850 nm) sterically stabilized by a chemically grafted poly-12-hydroxy stearic acid23 suspended in octadecence, chosen because it has a very low volatility. Suspensions were centrifuged at 6000rpm for 24 hours to create a sediment which we assume has a

volume fraction of φ0 ~ 0.64 (random close packing). Subsequent volume fraction solutions were prepared by dilution of this stock. After dilution and prior to use, the samples were thoroughly mixed with a vortex whirlie mixer. The sample was turned upside down and allowed to flow under the vibrations of the whirlie mixer to the end of the container. The sample was then inverted and the procedure repeated many times to ensure uniformity. We observed no granulation during this process with the sample surface remaining glossy. In removing sample from the vial and during loading the spatula was moved very slowly to prevent granulation. The sample was loaded into the instrument with the plates 3mm apart. The gap was then decreased to 2mm at a velocity of 1μm/s. After loading the samples were allowed to rest for 2 minutes before the experiment. The use of a 2mm gap was chosen as a compromise between minimising radial shear rate (which increases with 1/square of the gap) whilst still preventing the sample from seeping out from between the plates.

Rheometer Measurements:

Measurements were made using a Malvern Kinexus pro+ shear rheometer. The peak force was determined from the raw normal force data by spline fitting. The errors increased dramatically at higher velocities (>2mm/s) owing to noise from the instrument. Corrections due to instrument inertia were found to be smaller than the associated errors from other sources at all velocities. The maximum normal load measurable with our rheometer is limited to 20N which confines us to using small diameter plates (6mm) in continuity with our previous study [8]. A few measurements

were made with 8 and 4mm diameter plates, scaling the initial gap appropriately. We measured the same stresses as the 6mm case at the same strain rate.

The vibration stage:

The vibration stage consisted of a compression driver with a 6mm aluminium post mounted on the upper surface. The post protruded through a brass cylindrical hole which limited lateral movement of the post to < 0.2mm. A sinusoidal signal was applied to the compression driver via an amplifier. The vibrations were characterised using a 52-6000 accelerometer.
